# A nomogram based on conventional and contrast-enhanced ultrasound radiomics for the noninvasively prediction of axillary lymph node metastasis in breast cancer patients

**DOI:** 10.3389/fonc.2024.1400872

**Published:** 2024-05-10

**Authors:** Chao Sun, Xuantong Gong, Lu Hou, Di Yang, Qian Li, Lin Li, Yong Wang

**Affiliations:** ^1^ Department of Ultrasound, National Cancer Center/National Clinical Research Center for Cancer/Cancer Hospital, Chinese Academy of Medical Sciences and Peking Union Medical College, Beijing, China; ^2^ Department of Radiation Oncology, National Cancer Center/National Clinical Research Center for Cancer/Cancer Hospital, Chinese Academy of Medical Sciences and Peking Union Medical College, Beijing, China; ^3^ Department of Ultrasound, Affiliated Cancer Hospital of Zhengzhou University, Zhengzhou, China; ^4^ Department of Diagnostic Radiology, National Cancer Center/National Clinical Research Center for Cancer/Cancer Hospital, Chinese Academy of Medical Sciences and Peking Union Medical College, Beijing, China

**Keywords:** axillary lymph node, breast cancer, radiomics, conventional ultrasound, contrast-enhanced ultrasound

## Abstract

**Background:**

This study aimed to investigate whether quantitative radiomics features extracted from conventional ultrasound (CUS) and contrast-enhanced ultrasound (CEUS) of primary breast lesions can help noninvasively predict axillary lymph nodes metastasis (ALNM) in breast cancer patients.

**Method:**

A total of 111 breast cancer patients with 111 breast lesions were prospectively enrolled. All the included patients received presurgical CUS screening and CEUS examination and were randomly assigned to the training and validation sets at a ratio of 7:3 (n = 78 versus 33). Radiomics features were respectively extracted based on CUS and CEUS using the *PyRadiomics* package. The max-relevance and min-redundancy (MRMR) and least absolute shrinkage and selection operator (LASSO) analyses were used for feature selection and radiomics score calculation in the training set. The variance inflation factor (VIF) was performed to check the multicollinearity among selected predictors. The best performing model was selected to develop a nomogram using binary logistic regression analysis. The calibration and clinical utility of the nomogram were assessed.

**Results:**

The model combining CUS reported ALN status, CUS radiomics score (CUS-radscore) and CEUS radiomics score (CEUS-radscore) exhibited the best performance. The areas under the curves (AUC) of our proposed nomogram in the training and external validation sets were 0.845 [95% confidence interval (CI), 0.739-0.950] and 0.901 (95% CI, 0.758-1). The calibration curves and decision curve analysis (DCA) demonstrated the nomogram’s robust consistency and clinical utility.

**Conclusions:**

The established nomogram is a promising prediction tool for noninvasive prediction of ALN status. The radiomics features based on CUS and CEUS can help improve the predictive performance.

## 1 Introduction

Breast cancer is the most common cancer and the leading cause of tumor-related mortality in female patients worldwide (1). Though about 98.6% of breast cancer patients could survive for 5 years after the diagnosis, this rate would decrease to 84.4% in the presence of axillary lymph node metastasis (ALNM) (2, 3). Axillary lymph node (ALN) status is an independent prognostic indicator for disease-free survival and overall survival in early-stage patients with breast cancer (4). The correct preoperative staging of ALN status is of important clinical significance for the optimization of clinical decision.

Currently, axillary lymph node dissection (ALND) is the widely recognized method for identifying metastatic ALNs, which is invasive and associated with a series of complications including lymphedema, nerve injury, abnormal function (5, 6). To minimize unnecessary body damage, the sentinel lymph node biopsy (SLNB) has become the preferred approach for evaluating ALN status. However, SLNB is also accompanied with a series of side effects such as infection, allergies to tracer agents, skin staining, longer surgical times and higher surgical trauma (7–9).

Conventional ultrasound imaging (CUS) is a commonly recommended method for preoperative assessment of the ALN status, owing to its convenience, radiation-free and non-invasive advantages. However, the value of CUS in identifying ALNM is limited, with a sensitivity ranging from 48% to 87% and a specificity ranging from 55% to 97% (10). For the ALN involved with micro-metastasis, it will become imperceptible on CUS images. To tackle this issue, recent studies have attempted to exploit the ultrasonic features of primary breast lesions to predict ALNM (11–13). These ultrasonic features encompassed both the morphological information derived from CUS and the functional information derived from CEUS (11–13). However, the predictive performances based on these features are not excellent. The development of more effective assessment methods for noninvasive prediction of ALNM is imperative.

Radiomics is a widely applied technique for extracting high-throughput features from medical images. A great amount of high-dimensional features including shape, intensity and texture information that were unable to observe with the naked eye can be obtained and objectively analyzed using radiomics method (14, 15). Based on CUS images of primary breast tumor, ultrasound radiomics has been applied to establish prediction model for ALN status in several studies (16–18).

However, there is currently no study reporting the utility of radiomics features derived from CEUS in predicting ALN status in patients diagnosed with breast cancer. On the basis of routine CUS screening among breast cancer patients, it is convenient to perform CEUS and obtain the perfusion information regarding the primary breast lesions. In this study, we aimed to investigate whether quantitative radiomics features derived from both CUS and CEUS could help improve the predictive performance of ALNM.

## 2 Materials and methods

This was a single-center prospective study approved by the ethics committee of the cancer hospital of the Chinese Academy of Medical Sciences. The enrolled patients had given their informed consent to participate this study.

### 2.1 Patient selection and sample size estimation

**Figure 1:**
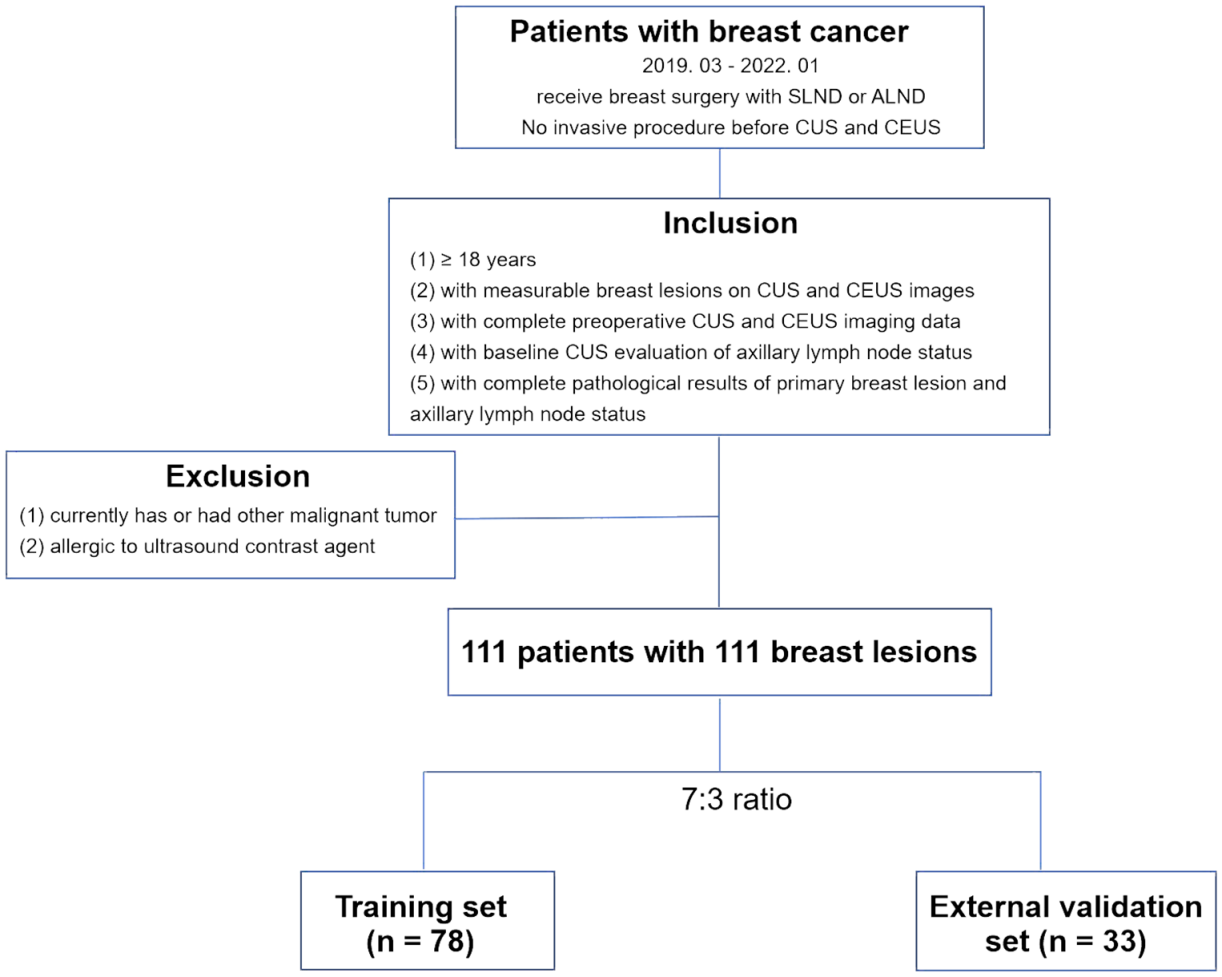
Flowchart for the patient selection criteria. CUS, conventional ultrasound; CEUS, contrast-enhanced ultrasound. SLNB, sentinel lymph node biopsy; ALND, axillary lymph node dissection.

From March 2019 to January 2022, a total of 111 breast lesions from 111 female patients were enrolled in this study based on the following inclusion criteria: (1) patients who were aged ≥ 18 and diagnosed with breast malignant cancers by postoperative pathology results. (2) with the presence of measurable lesions (≥1) proven by conventional ultrasound (CUS) and contrast enhanced ultrasound (CEUS) performed before any interventional treatment, including core needle biopsy, neoadjuvant chemotherapy, or surgery. (3) with complete baseline CUS images of breast lesions and ipsilateral axillary lymph node (ALN) assessment based on CUS features. (4) with complete baseline CEUS videos of breast lesions. (5) with clearly verified ALN status by pathology after sentinel lymph node biopsy (SLNB) or axillary lymph node dissection (ALND). The exclusion criteria were as follows: (1) currently has or had a history of malignant tumors besides breast cancer. (2) allergic to ultrasound contrast agents or other contraindication for ultrasound contrast agent application. The enrolled patients were assigned numbers and subjected to stratified random sampling based on the pathological status of ALNs in order to create a training set and an external validation set at a ratio of 7:3 (**Figure 1**).

The sample size estimation was based on the reported axillary lymph node metastasis (ALNM) incidence in breast cancer patients and the principle of 10 outcome events per variable (19–21). Using an estimated ALNM incidence of 0.45 in the study population and for three predictors, we aimed to enroll 67 breast cancer patients but actually enrolled 70 in the training set.

### 2.2 Clinical and pathological information collection

The clinical and pathological characteristics of the enrolled patients, including age, breast tumor size (measured by CUS), breast tumor histological type, pathological ALN status, breast tumor receptor (estrogen receptor, ER; progesterone receptor, PR; human epidermal growth factor receptor, Her-2) status, were collected from the medical records system. All the breast lesions were surgically removed, following by ALND. The breast tumor histology and pathological ALN status were documented according to the postoperative pathological results.

### 2.3 CUS examination procedure and CUS-reported ALN status

The CUS was performed by two senior sonographers (5 years’ experience in CUS diagnosis of breast tumor) using Philips EPIQ5 ultrasonic diagnostic equipment (Philips, Bothell, WA) with high frequency linear array probes to choose the target breast lesion and best sonographic sections for the observation. The whole breast underwent CUS screening and the identified breast lesions were graded based on the second edition of the American College of Radiology Breast Imaging Reporting and Data System for US (22). The breast lesion with BI-RADS grade 4C or BI-RADS grade 5 was considered as suspiciously malignant. The lesion size was measured on CUS images. If multiple breast lesions were suspiciously malignant in a patient, the biggest one was selected as the target lesion. The maximum transverse and longitudinal sections of each target lesion were respectively captured and stored, eventually obtaining two CUS images per target lesion. The ALN status was also evaluated by CUS. An ALN was defined as CUS-reported positive ALN when it presented one of the following features: irregular cortical thickness of greater than 3 mm, longest-to-shortest axis ratio less than 2, or absence of a fatty hilum (23). CUS images of target lesions and ALNs were initially stored in a Digital Imaging and Communications in Medicine (DICOM) format and subsequently converted to a Joint Photographic Experts Group (JPEG) format for further analysis.

### 2.4 CEUS examination procedure

The CEUS was also performed by two senior sonographers (5 years’ experience in CEUS diagnosis) using the same ultrasonic diagnostic equipment mentioned in CUS examination. First, the lyophilized powder of contrast agent (Sono Vue, Bracco SpA, Milan, Italy) was reconstituted by adding 5 mL of 0.9% saline and shaking to form a homogeneous microbubble suspension. Second, the real-time imaging of double-frame CEUS mode was activated after the proper ultrasonic section of the target lesion was displayed by CUS. Then, a bolus of 4.8-mL suspension of the contrast agent was administered via antecubital vein. The continuous storage of CEUS imaging and chronograph were initiated immediately following the injection of the contrast agent, lasting for a duration of 3 minutes. The CEUS imaging data was initially stored as a dynamic video with a DICOM format and subsequently converted to an Audio Video Interleaved (AVI) format for further analysis.

### 2.5 Region of interest segmentation

**Figure 2:**
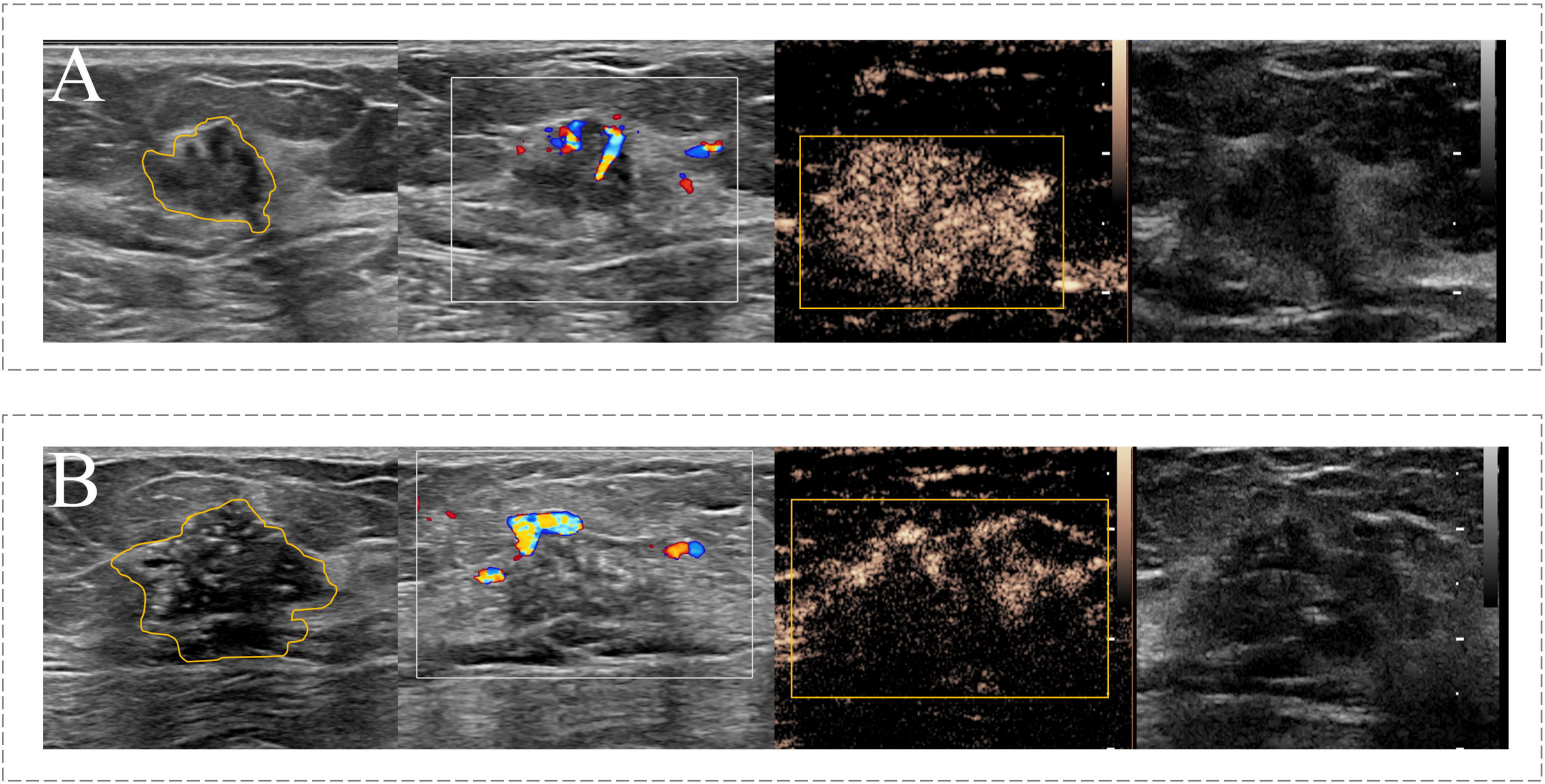
Example of the ROI segmentation in CUS image and CEUS videos. **(A)** The ROI segmentation on the CUS image (Left) and CEUS frame (Right) of malignant breast lesion with ALNM. **(B)** The ROI segmentation on the CUS image (Left) and CEUS frame (Right) of malignant breast lesion without ALNM.

Next, two CUS images (JPEG format) and one CEUS video (AVI format) of each target lesion was used for region of interest (ROI) segmentation. For CUS images, the ROI was delineated around the boundary of the target lesion. If the hyperechoic halo was present in CUS images, the boundary was positioned outside hyperechoic halo. For CEUS videos, a series of continuous frames of the CEUS video formed the CEUS images for target lesion. A rectangular box was outlined in the frame that can clearly displayed the tumor area and a computer vision algorithm was subsequently used to track and draw the ROI sketches ac nross the other frames in the video (24). The example of target lesion delineation based on CUS images and CEUS video frames were listed in **Figure 2**.

### 2.6 Feature extraction, selection and radiomics score calculation

The radiomics features were extracted from the ROI delineated based on CUS and CEUS images, respectively. An open-source *pyradiomics* package (http://github.com/Radiomics/pyradiomics) was used to extract shape features, first-order statistical features, and texture features from both the original images and transformed images, respectively. The transformed images were generated by performing 2D discrete wavelet decomposition and reconstruction, or filtering by the Laplacian of Gaussian method with different sigma parameters. Specifically, the shape features extracted from CEUS images were excluded due to the limitations of using a rectangular box delineation, which could not accurately capture the shape information of target lesion.

Next, the max-relevance and min-redundancy (MRMR) and least absolute shrinkage and selection operator (LASSO) analyses were used to respectively select the most effective CUS and CEUS feature subsets for the prediction of ALNM in the training set. Finally, two sets of radiomics scores based on CUS or CEUS images were respectively constructed with corresponding selected features.

### 2.7 Development and validation of nomogram

Before prediction model construction, the multicollinearity was analyzed by assessing the variance inflation factor (VIF) among involved variables. Multicollinearity was considered to exist when a VIF value was above 3. The clinical characteristic with P <0.05 in the univariate analysis was incorporating with radiomics scores based on CUS and CEUS images to construct a nomogram as a quantitative tool to predict ALNM using multivariable logistic regression analysis. The discriminatory ability of the model was evaluated using receiver operating characteristic (ROC) curve analysis and the area under curve (AUC) in the training data and validation date. The Delong algorithm was used to compare AUC of different models (P < 0.05). The predictive accuracy of the model was evaluated by calibration curve. A decision curve analysis was performed to determine the clinical usefulness.

### 2.8 Statistical analysis

R software (ver.1.4.1717, R Development Core Team) and SPSS 22.0 software (IBM Corporation, NY, USA) were used for statistical analysis. The *χ* ^2^ test or Fisher’s exact test were used for the comparison of classification variables, whereas the independent-sample *t* test was used for the comparison of continuous variables. A *P* value<0.05 was considered statistically significant. SPSS was used for binary logistic regression analysis and a series of packages in R software were used to develop the predictive model and test the diagnostic performance of the model. The corresponding packages included the rsample, mRMRe, glmnet, caret, corrplot, survival, ggplot2, rms, pROC, tidyverse, rmda and ggDCA packages.

## 3 Results

### 3.1 Baseline characteristics of training set and external validation set

A total of 111 patients with 111 breast malignant lesions were enrolled in this study from March 2019 to January 2022. **Table 1** summarizes the baseline clinical characteristics of 78 patients in the training set and 33 patients in the validation set. These baseline characteristics included the age of each enrolled patient, size, histology type and receptor status of each breast tumor, as well as the pathological status of ALNM. A total of 27 (34.6%) patients with ALNM were included in the training set and 12 (36.4%) patients with ALNM were included in the external validation set. Both sets had comparable ALN prevalence rate (p = 0.86). Additionally, there are no significant differences in the other baseline characteristics between two sets.

**Table 1:** Baseline characteristics of patients in training set and external validation set.

Characteristic	Training set (*n* = 78)	External validation set (*n* = 33)	P value
Age (year)	50.3 ± 10.76	53 ± 7.91	0.068
Pathological ALN status			0.86
Positive	27 (34.6%)	12 (36.4%)	
Negative	51 (65.4%)	21 (63.6%)	
Histological type			0.349
IDC/ILC/IDLC	69 (88.5%)	27 (81.8%)	
DCIS	9 (11.5%)	6 (18.2%)	
Receptor status			
ER			0.143
**+**	56 (71.8%)	28 (84.8%)	
**-**	22 (28.2%)	5 (15.2%)	
PR			0.112
**+**	55 (70.5%)	28 (84.8%)	
**-**	23 (29.5%)	5 (15.2%)	
Her-2			0.833
**-/+**	47 (60.3%)	20 (60.6%)	
**++**	17 (21.8%)	5 (15.2%)	
**+++**	14 (17.9%)	8 (24.2%)	
Primary tumor size (cm)	2.3 ± 1.41	2.3 ± 1.20	0.689
CUS-reported ALN status			
Suspicious	27 (34.6%)	14 (42.4%)	0.436
Unsuspicious	51 (65.4%)	19 (57.6%)	

ALN, axillary lymph node; IDC, invasive ductal carcinoma; ILC, invasive lobular carcinoma; IDLC, mixed invasive ductal and lobular carcinoma. DCIS, ductal carcinoma in situ; ER, estrogen receptor; PR, progesterone receptor. Her-2, human epidermal growth factor receptor 2; CUS, conventional ultrasound.

### 3.2 Radiomics features selection and radiomics score calculation

**Figure 3:**
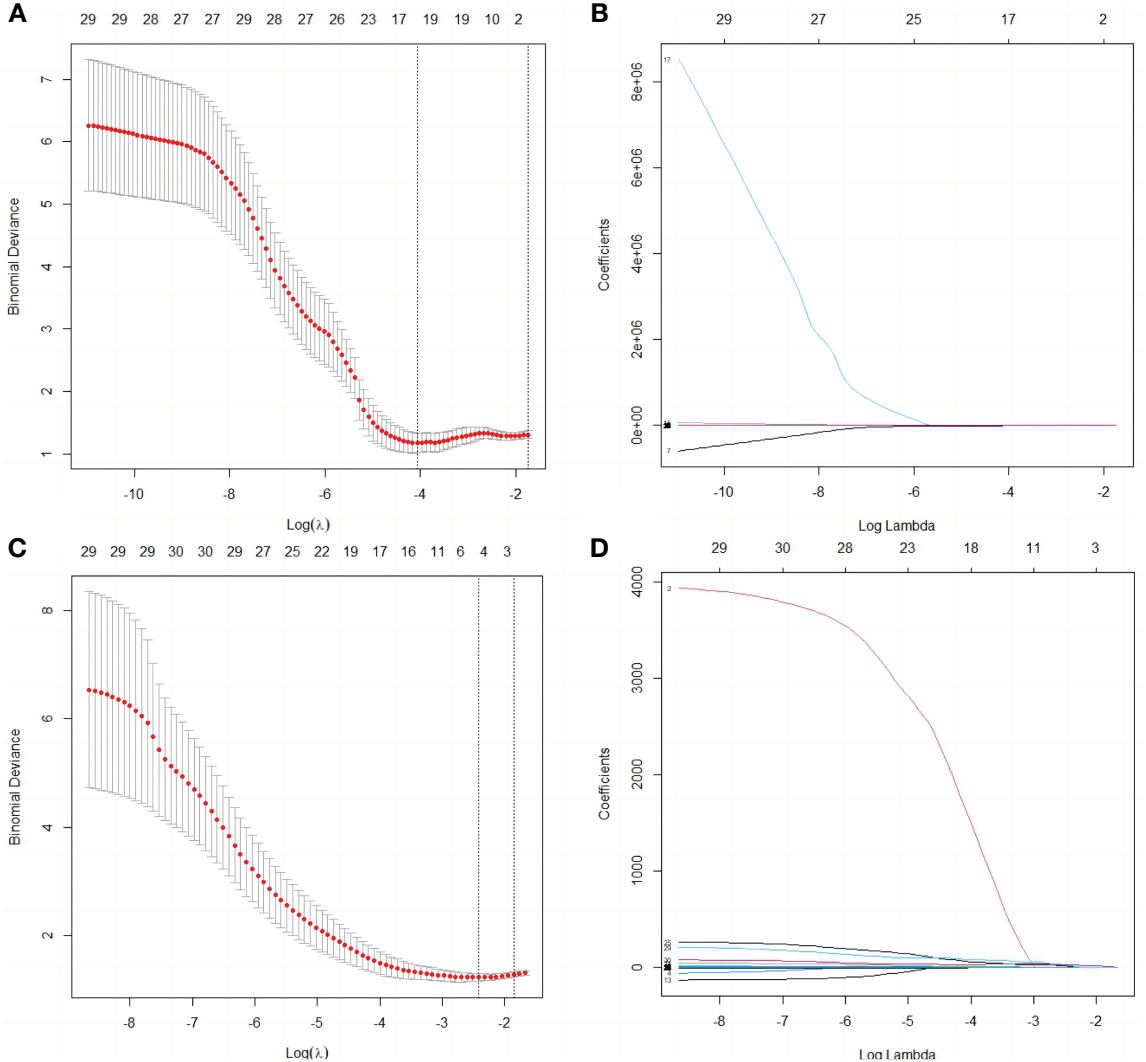
Selection of radiomics features by the LASSO analyses. **(A)** Selection of the tuning parameter λ in the LASSO analysis among candidate CUS radiomics features via 10- fold cross-validation based on the 1 standard error of the minimum criteria (1 – SE criteria). The value of λ that derived the minimum average binomial deviance was used to select features. Dotted vertical lines were drawn at the values using the minimum criteria and the 1 – SE criteria. **(B)** LASSO coefficient profiles of the 30 candidate CUS radiomics features. **(C)** Selection of the tuning parameter λ in the LASSO analysis among candidate CEUS radiomics features via 10- fold cross-validation based on the 1 standard error of the minimum criteria (1 – SE criteria). The value of λ that derived the minimum average binomial deviance was used to select features. Dotted vertical lines were drawn at the values using the minimum criteria and the 1 – SE criteria. **(D)** LASSO coefficient profiles of the 30 candidate CEUS radiomics features.

A total of 1530 radiomics features (CUS-rad-features) were extracted from CUS images, while 1395 radiomics features (CEUS-rad-features) were extracted from CEUS images. Next, top 30 features were respectively selected from these CUS-rad-features and CEUS-rad-features using MRMR algorithm. Finally, two CUS-rad-features and two CEUS-rad-features with non-zero coefficients were respectively identified by LASSO regression model (**Table 2**, **Figure 3**). The radiomics scores based on CUS images and CEUS images were respectively calculated using the final selected features to generate CUS-radscore and CEUS-radscore for model construction.

**Table 2:** List of the selected radiomics features extracted from CUS and CEUS images via LASSO analyses.

Image source	Image Type	Feature Class	Feature Name	Coefficient
CUS	Log_sigma	GLSZM	GrayLevelNonformity	0.0014277
CUS	Log_sigma	firstorder	InterquartileRange	-0.027179
CEUS	Wavelet_LH	GLCM	ClusterProminence	0.0134133
CEUS	Wavelet_LL	GLSZM	SmallAreaLowGrayLevel Emphasis	8.9129928

CUS, conventional ultrasound; CEUS, contrast enhanced ultrasound. GLSZM, Gray Level Size Zone Matrix; GLCM, Gray Level Co-occurrence Matrix.

### 3.3 Development and validation of the nomogram

**Figure 4:**
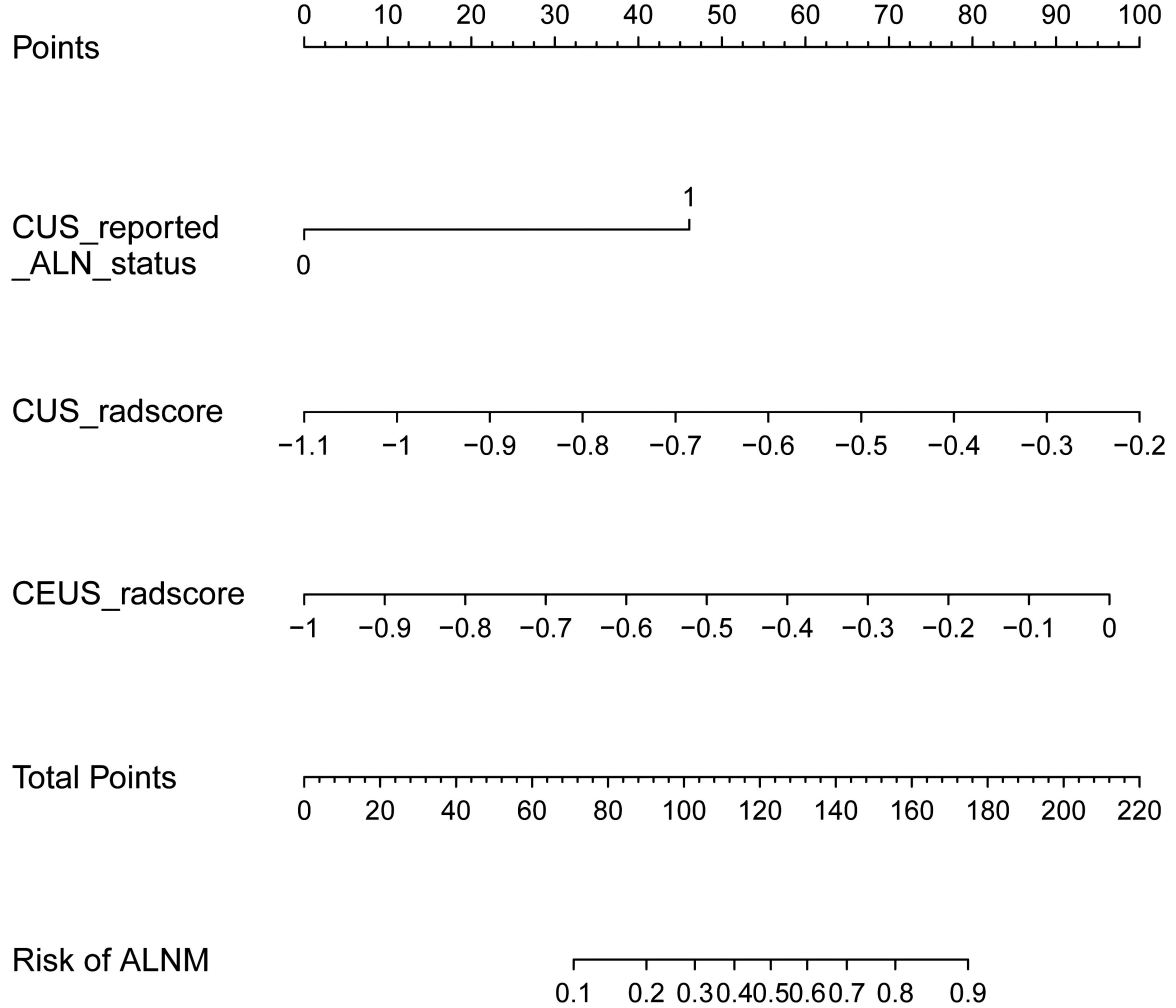
The nomogram developed in the training set using CUS-reported ALN status, CUS-radscore, and CEUS-radscore as predictors. The nomogram plot provides a visual way to calculate the risk of ALNM for breast cancer patients.

**Figure 5:**
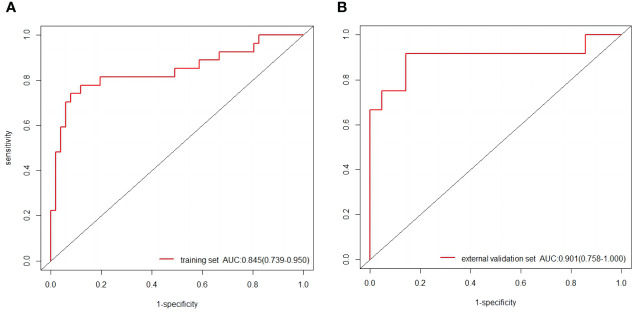
**(A)** The ROC curves of the nomogram in the training set. **(B)** The ROC curves of the nomogram in the external validation set.

The baseline characteristics that were accessible prior to the surgical operation included age, primary tumor size and CUS-reported ALN status. The multivariate analyses further showed that CUS-reported ALN status was statistically related to ALNM (**Table 3**). Thus, a total of three predictors including CUS-reported ALN status, CUS-radscore and CEUS-radscore were obtained. The VIFs of these three predictors ranged from 1.269 to 1.439, indicating no multicollinearity existed among them. Next, three prediction models were respectively established by incorporating different amounts of predictors using logistic regression analysis (Model 1: CUS-reported ALN status; Model 2: CUS-reported ALN status + CUS-radscore); Model 3: CUS-reported ALN status + CUS-radscore + CEUS-radscore). The AUC values of three models in both training and external validation set were summarized (**Table 4**). Model 3 showed better performance than model 2 or model 1 in both the training set (AUC: 0.845 vs. 0.826 or 0.773, P = 0.4581 and P < 0.01) and external validation set (AUC: 0.901 vs. 0.889 or 0.821, P = 0.738 and P = 0.0283). Additionally, a better performance was also observed by adding CUS-radscore on the basis of CUS-reported ALN status in the training set (AUC: 0.826 vs. 0.773, P < 0.001) and the external validation set (AUC: 0.889 vs. 0.821, P = 0.013).Therefore, a nomogram was constructed using model 3 (**Figure 4**). The ROC curves of both the training and external validation set all showed excellent results (**Figure 5**). By incorporating three predictors, model 3 yielded an AUC value of 0.845 [95% confidence interval (CI), 0.739-0.950] with a sensitivity of 0.74.1% and a specificity of 92.2% in the training set, and an AUC of 0.901 (95% CI, 0.758-1) with a sensitivity of 91.7% and a specificity of 85.7% in external validation set.

**Table 3:** The multivariate logistic analysis to identify independent predictor for ALNM among baseline characteristics in the training set.

Variables	Odds ratio	95% CI	P value
Age	1.026	0.967-1.088	0.403
Primary tumor size	1.496	0.986-2.270	0.058
CUS-reported ALN status (Suspicious)	12.719	3.808-42.489	< 0.01

ALNM, axillary lymph node metastasis; CUS, conventional ultrasound; CI, confidence interval.

**Table 4:** Performance of different models in training and external validation set.

Model	Training set		External validation set	
	AUC	95% CI	AUC	95% CI
1	0.773	0.672-0.875	0.821	0.682-0.961
2	0.826	0.719-0.932	0.889	0.750-1.00
3	0.845	0.739-0.950	0.901	0.758-1.00

Model 1, the model incorporating CUS-reported ALN status alone as the predictor; Model 2, the model incorporating CUS-reported ALN status and CUS-radscore as the predictors. Model 3, the model incorporating CUS-reported ALN status, CUS-radscore and CEUS-radscore as the predictors; AUC, area under curves; CI, confidence interval.

**Figure 6:**
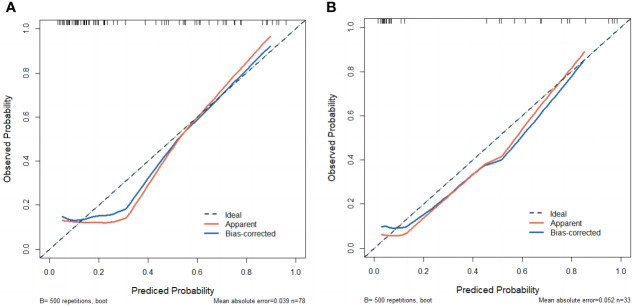
**(A)** The calibration curve of the nomogram in the training set. **(B)** The calibration curve of the nomogram in the external validation set.

**Figure 7:**
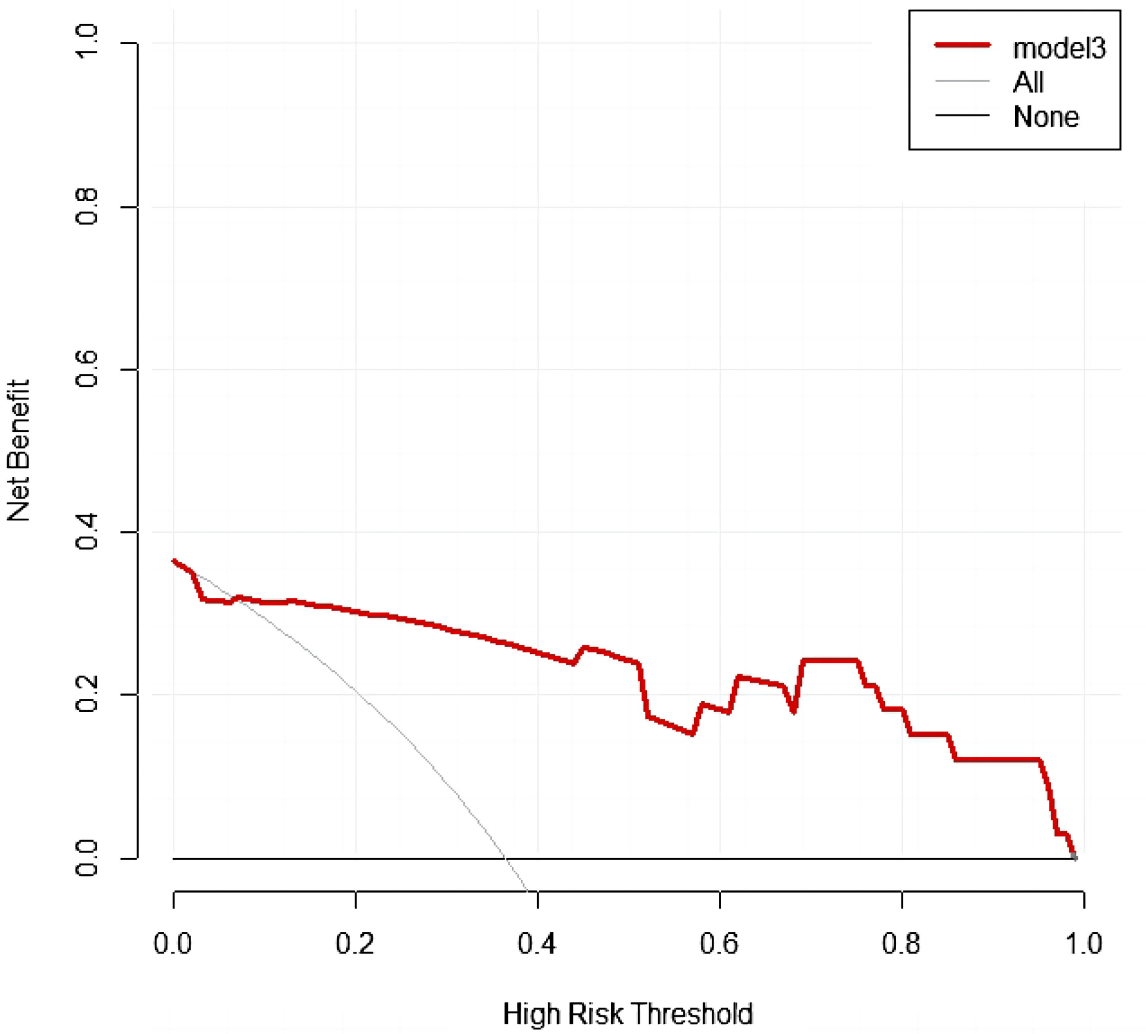
Decision curve analysis in external validation set.

Calibration curves of the nomogram based on the training and external validation set were plotted to evaluate the consistency between the predicted probability of ALNM and actual pathological results of ALN (**Figure 6**). The calibration curves of our established nomogram showed a good fitting with the ideal curve in both training and external validation set. The decision curve analysis displayed a positive net benefit for the nomogram when a threshold probability was greater than 0.1, indicating a good clinical utility (**Figure 7**).

### 3.4 Representative examples of the nomogram in clinical practice

**Figure 8:**
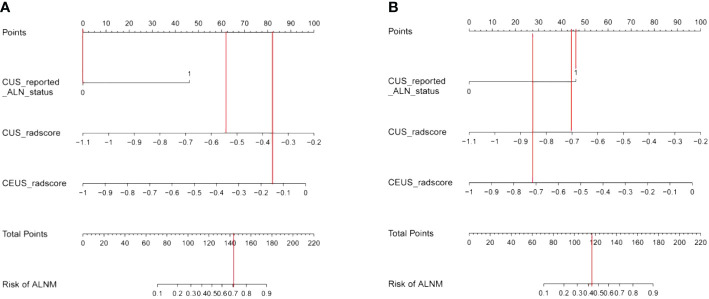
**(A)** Examples of nomogram evaluation of ALNM in patients with false-negative CUS findings. **(B)** Examples of nomogram evaluation of ALNM in patients with false-positive CUS findings.

Then, we listed two examples of the clinical practice of our proposed nomogram. Patient 1, a 46-year-old woman, was assessed without ALNM by CUS, while an ALNM probability over 0.7 was calculated by the nomogram (**Figure 8A**). Pathology results confirmed metastatic ALNs were present in this patient. Patient 2, a 49-year-old woman, was assessed with ALNM by CUS, while an ALNM probability around 0.42 was calculated by the nomogram (**Figure 8B**). Pathology results confirmed metastatic ALNs were absent in this patient.

## 4 Discussion

In this study, we developed and validated a nomogram to predict ALNM by combining CUS-reported ALN status and radiomics features that were derived from both CUS and CEUS images. A good performance was achieved by our proposed nomogram, with an AUC of 0.845 in the training set and 0.901 in the external validation set.

The ALN status holds significant prognostic value for breast cancer. For invasive breast cancer accompanied with ALNM, ALND is an important treatment option in clinical practice. However, ALND may not suitable for all the patients with breast cancer due its associated complications (5, 6). SLN serves as the first station for lymph node metastases of primary tumors. SLND is considered as an alternative to ALND to determine the ALN status. For SLN negative patients, ALND is not recommended (25, 26). However, SLND is also associated with potential side effects and longer operation time, as well as a certain rate of false negative (7–9, 27). In the background of precision medicine, it is urgent to more effective method for uninvasive prediction of ALNM.

CUS is the routine method for noninvasive assessment of ALN status in breast cancer patients. The presence of irregular cortical thickness, longest-to-shortest axis ratio less than 2, and the absence of a fatty hilum were considered as ultrasonic features suggestive for ALNM on CUS images (23). However, it is difficult for CUS to achieve high accuracy depend on these CUS features for identifying ALNM, especially in pathological N1 patients (10, 28). Recently, several studies have found that the CUS features of primary breast lesions, including tumor size, margin, location and echogenicity were correlated with the tumor biological behavior and thus can help predict ALN status (29–33). Additionally, other studies have reported a potential association between CEUS findings and the prognosis of breast cancers (34, 35). CEUS is also a noninvasive imaging modality that can be conveniently performed on the basis of CUS screening. Different from contrast enhanced CT or MR imaging, CEUS used true intravascular contrast agents without deposition into extravascular space and was capable of reflecting the micro-vascular distribution. In common, the proliferation of vessels would drive the aggressive growth of tumors (36). It was reported that coarse or twisted vessels and enhanced range of the primary tumors were independent predictors for metastatic ALNs (12, 13). The findings of these studies provide support for the feasibility of utilizing radiomics features based on CUS and CEUS to predict the ALN status.

Radiomics can objectively extract and quantitatively analyze features from medical images. In this study, radiomics features were separately extracted from CUS images and CEUS videos. An auto-tracer ROI segmentation technology was applied to outline target region on a continuous series of frames of CEUS video for each target lesion (24). Specifically, Gray Level Size Zone GLSZM (GLSZM) feature was selected as the preferred option among either CUS-radiomics or CEUS-radiomics features. GLSZM measures the size of homogeneous zones for each gray level in an image (37). Previous study demonstrated CLSZM was the optimum texture feature for breast lesion characterization (37, 38). The feature selection results obtained in our study have demonstrated that GLSZM is also optimum feature for the predicting ALNM. It is worth noting that the finally selected CEUS-radiomics features were all generated from images after wavelet transformation. The wavelet transformation is a mathematical algorithm which can mine the hidden patterns from various data, which is not visible to the naked eye (39). Compared with applying CUS-reported ALN status alone, the introduction of either CUS-radiomics features (CUS-radscore) or combined radiomics-features (CUS-radscore and CEUS-radscore) significantly increased the predictive performance. Furthermore, the combination of CUS-radscore, CEUS-radscore and CUS-reported ALN status achieved the highest AUC value, demonstrating the potential value of CEUS for ALNM prediction. Despite an improvement in AUC values for either the training set or external validation set was observed after the incorporation of the CEUS-radscore, the result of Delong test did not reveal a significant difference in AUC values between model 2 (CUS-reported ALN status + CUS-radscore) and model 3 (CUS-reported ALN status + CUS-radscore + CEUS-radscore).This could potentially be attributed to the relatively limited sample size in this study and the N stage of enrolled patients. Given that CUS-reported ALN status exhibits lower diagnostic efficacy in pathological N1 patients (10, 28), integrating predictors derived from CUS or CEUS radiomics into constructing a predictive model holds greater potential for improving AUC values among pathological N1 patients. However, the relatively small sample size of this study restricted the screening of pathological N1 patients for further analysis. A multi-center study with a larger sample size would help to better elucidate the value of CEUS-radscore in predicting ALNM. In clinical practice, the proposed nomogram in our study provides a practical method to quantitatively evaluate the ALNM probability among breast cancer patients. The ALNM probability calculated by the nomogram can serve as a valuable reference for clinicians in determining the necessity of implementing ALND.

There are some limitations of this study that should be mentioned. First, as we mentioned above, the sample size is relatively small and a multi-center study with larger sample size need to be further implemented. Second, a rectangular box was outlined for the ROI segmentation based on CEUS videos. Thus, the shape features derived from CEUS videos could not be used as candidate features. Third, certain ultrasonic sections for target lesions were used to present the whole 3-dimensional lesion for radiomics feature extraction. Fourth, the inter-observer and intra-observer agreement during ROI delineation was not assessed in this study.

## 5 Conclusions

This study has established and validated a nomogram for the prediction of ALNM in patients with breast cancer. On the basis of CUS-reported ALN status, the introduction of CUS and CEUS radiomics features derived from primary breast lesions can further improve the predictive performance. Our proposed nomogram is very important in guiding clinical decision and avoid unnecessary invasive operation.

## Data availability statement

The raw data supporting the conclusions of this article will be made available by the authors, without undue reservation.

## Ethics statement

The studies involving humans were approved by the ethics committee of the Cancer Hospital, Chinese Academy of Medical Sciences and Peking Union Medical College. The studies were conducted in accordance with the local legislation and institutional requirements. Written informed consent for participation in this study was provided by the participants’ legal guardians/next of kin.

## Author contributions

CS: Writing – original draft, Methodology, Formal analysis, Conceptualization. XG: Writing – review & editing, Methodology, Data curation. LH: Writing – review & editing, Software, Methodology. DY: Writing – review & editing, Resources, Investigation, Conceptualization. QL: Writing – review & editing, Methodology. LL: Writing – review & editing. YW: Writing – review & editing, Supervision, Funding acquisition, Conceptualization.
